# Regulation of the meiotic divisions of mammalian oocytes and eggs

**DOI:** 10.1042/BST20170493

**Published:** 2018-06-22

**Authors:** Jessica R. Sanders, Keith T. Jones

**Affiliations:** School of Biological Sciences, Faculty of Natural and Environmental Sciences, University of Southampton, Southampton SO17 1BJ, U.K.

**Keywords:** cell cycle, female gametes, meiosis

## Abstract

Initiated by luteinizing hormone and finalized by the fertilizing sperm, the mammalian oocyte completes its two meiotic divisions. The first division occurs in the mature Graafian follicle during the hours preceding ovulation and culminates in an extreme asymmetric cell division and the segregation of the two pairs of homologous chromosomes. The newly created mature egg rearrests at metaphase of the second meiotic division prior to ovulation and only completes meiosis following a Ca^2+^ signal initiated by the sperm at gamete fusion. Here, we review the cellular events that govern the passage of the oocyte through meiosis I with a focus on the role of the spindle assembly checkpoint in regulating its timing. In meiosis II, we examine how the egg achieves its arrest and how the fertilization Ca^2+^ signal allows the initiation of embryo development.

## Introduction

In mammals, under hormonal cues, oocytes mature into eggs that are then fertilized [1–3]. Oocytes spend the vast majority of their life having entered meiosis but arresting before its completion. In the fetal ovary, oocytes are formed from primordial germ cells. These cells commit in a one-way journey to enter meiosis, which at its earliest stage, involves a whole genome replication (S-phase) followed immediately by a specialized pairing of all homologous chromosomes. In this way, a newly replicated pair of maternal origin chromosome 1 joins up with a newly replicated paternal origin chromosome 1, and so on for all autosomes as well as the sex chromosomes that pair through their PAR regions. In what appears to be the biological pinnacle of self-harm, these paired homologous chromosomes are then fragmented by deliberate double-strand DNA breakage [4–6]. The subsequent repair of such breaks results in a physical tethering of the two pairs of homologous chromosomes and generates chromosomes made from both maternal and paternal components that will eventually separate. Therefore, the self-harm is indeed essential given it provides a shuffling of the genes that is so advantageous and which, from an evolutionary perspective, gives meaning to sexual reproduction.

The physically tethered pair of homologous chromosomes (homolog pairs) forms a structure known as a bivalent. In the two meiotic divisions that follow, the bivalent divides reductionally in meiosis I (MI) to form two sister chromatid pairs. Cell division is very asymmetric in oocytes during MI, such that the products are a mature egg and a much smaller first polar body (PB1), which later degenerates and plays no part in embryo development. In mammals, it is a hormonal cue, luteinizing hormone (LH), which is responsible for this process. Prior to this signal, the oocyte is essentially in an arrested dictyate state, and also said to be in the germinal vesicle (GV) stage which is similar to G2 of a somatic cell. As such, as the oocyte resumes MI, it undergoes a process of nuclear envelope breakdown (NEB) caused by activation of Cdk1 — this is a G2/M transition [7,8]. In mammals, the time from a rise in LH to PB1 extension is several hours but varies between species [9]. Luckily for research purposes such a process, known as oocyte (or meiotic) maturation, can be replicated simply by removing the oocyte from the ovary and culturing it in media. This is because the ovary provides an oocyte maturation inhibitory environment [10].

Having completed MI, the mature egg rearrests at metaphase of meiosis II (MII). Such an event happens a short while before ovulation. This means that a metaphase II (metII)-arrested egg is ovulated into the female reproductive tract. If sperm are present, the egg is fertilized and it is this event that triggers the egg to complete MII. This second division, which is also asymmetric, resulting in a one-cell embryo and a second polar body (PB2) is described as an equational division. It involves the segregation of a sister chromatid pair and, in this respect, it resembles chromosome division in mitosis.

This review focuses on how the two meiotic divisions of the mammalian oocyte, specifically mouse oocytes, are controlled. It begins with an examination of how bivalents congress on the meiotic spindle in MI during oocyte maturation followed NEB; and it explores the factors responsible for faithful segregation. It then examines how metII is achieved and how sperm break this arrest from the perspective of cell division. We make analogy and give contrast where needed between these two divisions that temporally are separated by only a few hours.

## Meiosis I completion

**Figure 1.**
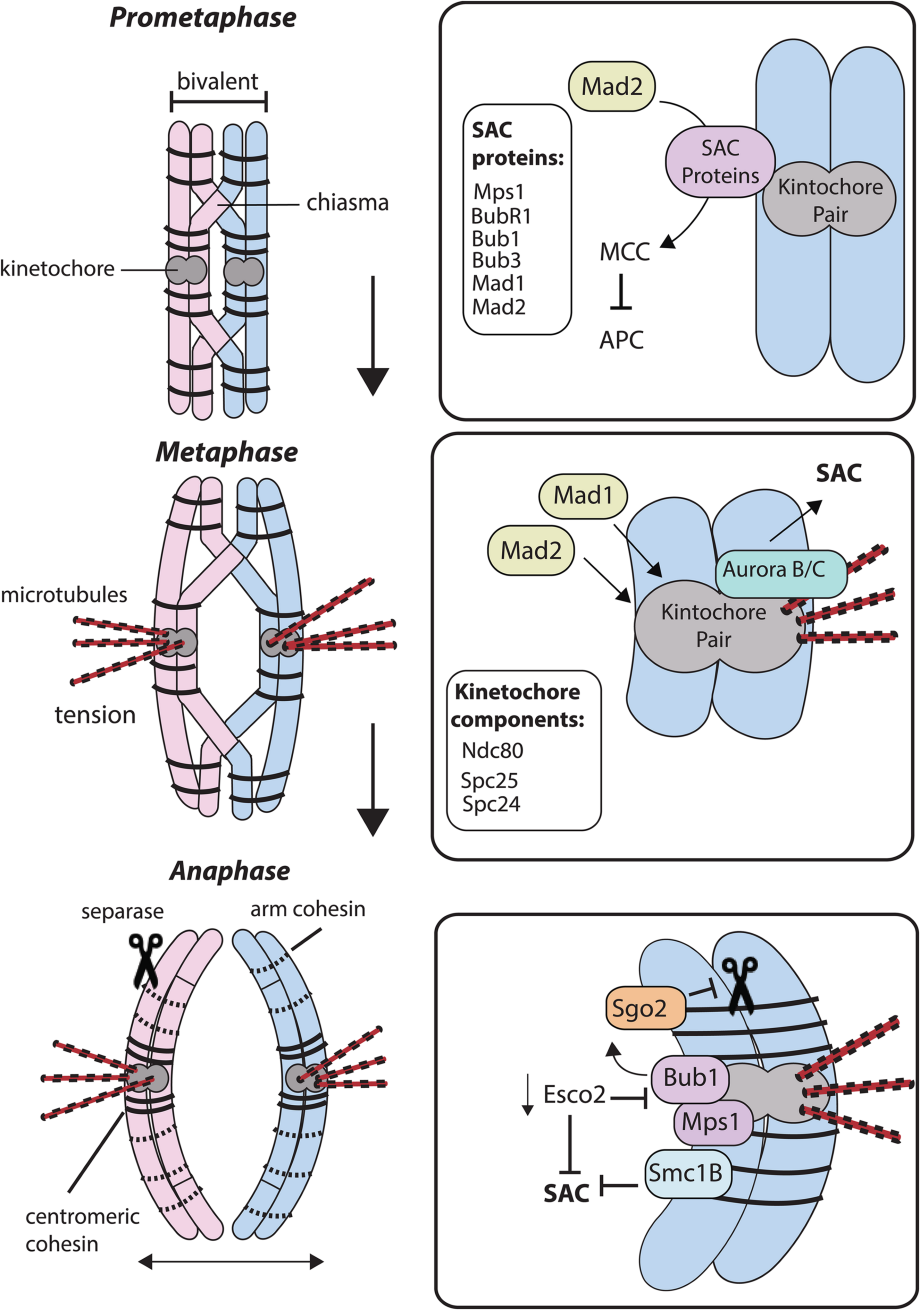
Cell cycle proteins mediating progression during MI. Homologous chromosome pairs are bound together through recombination-derived cross-over events, forming a bivalent. During prometaphase and metaphase, the SAC is active due to a lack of microtubule–kinetochore attachment and/or tension between the bivalent and microtubules. During SAC activation, Mps1 is recruited to unattached kinetochores leading to further recruitment of Bub1, Bub3 and BubR1. Mad1 and Mad2 are also directly recruited to the kinetochore. Mad2 undergoes a conformational change, which promotes the formation of the potent APC inhibitor MCC in the cytoplasm. The kinetochore protein Ndc80 (Hec1) binds directly to microtubules to promote microtubule–kinetochore attachment and also the recruitment of Mad1 to kinetochores. Spc24 and Spc25 are other kinetochore proteins which regulate SAC activation through the recruitment of Mad2. As microtubules attach to kinetochores correctly, tension across the bivalent increases. Insufficient tension can also activate the SAC via Aurora B/C activation and the error correction pathway. At anaphase, the bivalents are separated by the cleavage of cohesin by separase, but centromeric cohesin holding the chromatids together is protected. Sgo2 inhibits separase activity at the centromeric region. Mps1 and Bub1 ensure the correct localization of Sgo2. Esco2 is a cohesin-producing enzyme which is also essential for SAC activation. If Esco2 activity is not sufficient, SAC is not activated when required therefore proteins such as Bub1 are mislocalized. The cohesin subunit Smc1B is also necessary for SAC activation.

In mitosis, the kinetochores of sister chromatids bi-orientate towards opposite poles of the spindle but during MI the sister kinetochores align side-by-side, and in so doing ‘mono-orient’ towards the same pole. For mammalian oocytes, MI prometaphase is prolonged, lasting several hours, which may be due to this unique co-alignment of sister kinetochore pairs. Such an arrangement may well be inefficient in binding microtubules and achieving correct orientation with respect to the meiotic spindle. As with all other mammalian cell divisions, the segregation of chromosomes in MI is a result of anaphase-promoting complex (APC) activity which must be tightly controlled [11–15]. One component that regulates APC activity is the spindle assembly checkpoint (SAC). This brake acts as a block to anaphase until bivalents are captured on the spindle correctly and become aligned [16–20] (see Figure 1). The SAC is therefore thought to function in decreasing the risk of chromosome segregation errors. In oocytes, this is important given the high incidence of aneuploidies reported to occur that are derived from segregation errors in MI [21–25].

The meiotic SAC does not operate as an on/off binary switch [26] (in fact in mitosis, this is likely also to be the case [27]). As such, in maturing oocytes, the SAC is never truly off, such that even at the height of its activity the APC is only 50% of its possible maximal rate. 100% full APC is uncovered only in the presence of SAC inhibitors. Additionally, the switch from ‘off’ to ‘50% on’ is not entirely achieved through bi-orientation of bivalents, as oocytes can and do show considerable APC activity when bivalents are highly scattered in the cytoplasm [28]. The lack of ability of the SAC to act as a brake for completion of MI when bivalents are not properly bi-oriented has given it the moniker of being weak in oocytes. Indeed, it is now a well-established observation that oocytes can proceed through MI with a degree of bivalents not being bi-oriented [28–32]. However, their ability to respond well to DNA damage (see later) makes this observation surprising. The SAC can act as a strong brake in oocytes, so its strength appears dependent on what is the underlying trigger for its activation.

When the SAC is active, unattached kinetochores act as a docking site for proteins which bind in a hierarchal manner (see Figure 1). This is the case for both mitosis and oocytes in MI. During mitosis, Mps1 binds to free kinetochores and acts as a platform for binding other SAC proteins including Bub1, BubR1 and Bub3 [33]. Mps1 also binds kinetochores in oocytes and is essential for ensuring the correct timing of cell cycle progression [34]. Mps1 activity can be inhibited by the small molecule Reversine by ATP competition [35]. Bub1, BubR1 and Bub3 have all been found to play a role in regulating the SAC in mouse oocytes [36–38]. Mad1 and Mad2 are also expressed in mouse oocytes and are recruited directly to the kinetochores [39–41]. In mitosis, it is known that Mad2 undergoes a conformational change, resulting in the formation of a cytoplasmic mitotic checkpoint complex (MCC) that acts as a potent APC inhibitor [42]. Closed Mad2 (C-Mad2) with Mad1 binds directly to the kinetochore and recruits open Mad2 (O-Mad2), converting O-Mad2 to an intermediate form I-Mad2. This I-Mad2 binds to the APC activator cdc20 and closes thus inhibiting APC activity [43]. The activity of MCC during meiosis is not yet clear; however, it is very likely that there is a similar mechanism in oocytes because of the existence of its component parts (see Figure 1). Bisected oocytes that are free of bivalents rapidly lose this MCC brake to the APC, and so similar to somatic cells, it must be that the MCC is short-lived and requires the kinetochores for its continued existence [44]. It is unclear how far the MCC is able to diffuse in the cytoplasm of oocytes. As oocytes have a large volume, it is possible that the diffusible signal of the SAC response is not able to diffuse sufficiently which could lead to the weak SAC in oocytes [45]. However, even oocytes with over 80% reduced volume are still prone to chromosome segregation errors [46], so volume *per se* is unlikely to be an explanation of mis-segregation errors.

The loss of SAC components in oocytes leads to an acceleration through MI, principally because the brake is removed from APC activity anaphase happens sooner [21,25]. A high rate of bivalent mis-segregation leading to aneuploid eggs follows the loss of individual SAC components, which suggests that this checkpoint serves a necessary component in maintaining a high rate of faithful chromosome division in MI [34,36,37,40,47]. The SAC is sensitive to many different chromosome and spindle abnormalities in oocytes. One important factor in ensuring correct chromosome segregation is sufficient kinetochore–microtubule attachment. During mitosis, the KNL-1/Mis12 complex Ndc80 complex (KMN) network is responsible for promoting kinetochore–microtubule attachment and is composed of many protein complexes [48]. For example, Ndc80 (HEC1) binds directly to microtubules and anchors these to other protein complexes at the kinetochore [49,50]. Ndc80 is also responsible for recruiting SAC-related proteins such as Mad1 [51]. It is unclear whether the KMN network plays a role in monitoring kinetochore–microtubule attachments during meiosis; however, Ndc80 levels at microtubules increase during oocyte maturation and overexpression of Ndc80 causes cell cycle arrest in oocytes [52] (see Figure 1). Other components of the Ndc80 complex, Spc25 and Spc24, have also been found to play a role in regulating the SAC in oocytes including the recruitment of Mad2 [53,54] (see Figure 1). Other proteins associated with SAC activation such as Aurora B kinase may also play a role in stabilizing kinetochore–microtubule attachments [49,55,56]. During oocyte maturation, Aurora C kinase is also expressed and its activity appears to compensate for loss of Aurora B kinase [57]. Inhibiting Aurora kinase has been found to accelerate oocyte maturation and reverse an SAC-induced arrest [47]. A recent study has also suggested that Aurora B/C could play a role in detecting tension between bivalents and the spindle poles during MI. In this instance, Aurora B/C induces an error correction pathway that in turn activates SAC [58] (see Figure 1). This is a mechanism similar to that present for detecting insufficient chromatid tension during mitosis [59].

At chromosome segregation, the cohesin-binding homologous chromosomes together must be cleaved. This occurs as a result of separase activity following the activation of the APC [11]. However, because chromosome segregation in oocytes occurs in two phases, during MI and MII, it is key that the sister chromatids remain bound by centromeric cohesin and only the two homologous chromosome pairs that constitute the bivalent are separated during MI. Therefore, cohesin has to be removed in a stepwise manner. One protein involved in regulating this is Shugoshin 2 (Sgo2), which acts to protect centromeric cohesin from separase activity during MI through the recruitment of the PP2A phosphatase PP2A-C [60,61] (see Figure 1). It is thought that the correct localization of PP2A-C prevents separase cleavage of cohesin by dephosphorylating cohesin components such as Rec8 [61]. Interestingly, the correct localization of Sgo2 to the centromeric and pericentromeric region is dependent on the SAC components Mps1 and Bub1 [62] (see Figure 1). Insufficient sister chromatid cohesion could be an activator of the SAC in oocytes. Esco1 and Esco2 both play a role in regulating cohesin through the acetylation of the cohesin subunit Smc3. However, unlike Esco1, which is mainly responsible for maintaining the non-cohesive properties of cohesin, Esco2 plays a key role in establishing the cohesin complex and also in activating SAC [63]. Depletion of Esco2 leads to an inactivated SAC, loss of Bub1 from kinetochores and escape from MI arrest induced by the spindle poison nocodazole [64] (see Figure 1). The meiosis-specific cohesin subunit Smc1B is essential for maintaining sister chromatid cohesion through MI [65]. The presence of Smc1B is required for SAC activation and without it the chromosomes fail to align correctly [66] (see Figure 1). Securin, which binds and in so doing inhibits separase activity, is destroyed by APC during MI exit and can be modulated by the activity of SAC kinases [67]. When this centromeric cohesion is lost during MI, the SAC is not correctly activated and SAC-related proteins, such as Bub1, do not become correctly localized to the kinetochores [68] (see Figure 1).

Once the checkpoint has been satisfied, SAC proteins detach from the kinetochores and APC activity rises, albeit in oocytes to a submaximal peak [69]. During MI in oocytes, the SAC appears to be weak and ineffectual at detecting chromosomal alignment abnormalities compared with its activity during mitosis. As such, the SAC often allows the completion of meiosis even if not all the bivalents are correctly aligned or attached [70]. This leads to high rates of aneuploidy in oocytes by incorrect segregation of bivalents, raising the question of whether the SAC is activated/inactivated in response to any other factors during MI.

It appears that the SAC may play an additional role in responding to DNA damage accumulated during MI. For example, if DNA damage is induced in oocytes, the SAC is activated and the cell cycle is arrested at metaphase I (metI) [71,72]. This metI arrest can be triggered in response to DNA damage induced in a chemical or physical way but not by all types of DNA damage [73]. Double-strand breaks (DSBs) induced by neocarzinostatin, etoposide or laser beam dissection all increase the rate of MI arrest [73–75]. However, in contrast, interstrand cross-links induced by mitomycin C do not affect meiotic progression [75]. Interestingly, even though metI arrest occurs in oocytes exposed to DNA-damaging agents at any stage of MI, NEB still occurs in GV oocytes that have been exposed [73]. This suggests that there is no DNA damage checkpoint in operation in GV stage oocytes that prevents NEB. This is in contrast with somatic cells, where there is often a block in the G2/M transition following exposure to DSB-inducing agents [76]. The mechanism by which DSBs activate the SAC in oocytes is not yet fully known. It appears that DNA damage has no effect on kinetochore–microtubule interaction, the number of k-fibres, bi-orientation of the chromosomes or the tension across these chromosomes [73,77]. However, there is an assembly of SAC-associated proteins at the kinetochores [77]. Furthermore, this response appears to be reliant on the activity of Mps1, Aurora kinase and haspin [77]. Crucially, this DNA damage response does not involve Ataxia telangiectasia-mutated (ATM) or ATM and RAD3-related (ATR) signaling, which are normally integral to signaling in DNA damage responses [77]. Also, this arrest appears to be a unique feature of MI oocytes, as DNA damage in metII eggs does not activate the SAC [77].

## Meiosis II completion

**Figure 2.**
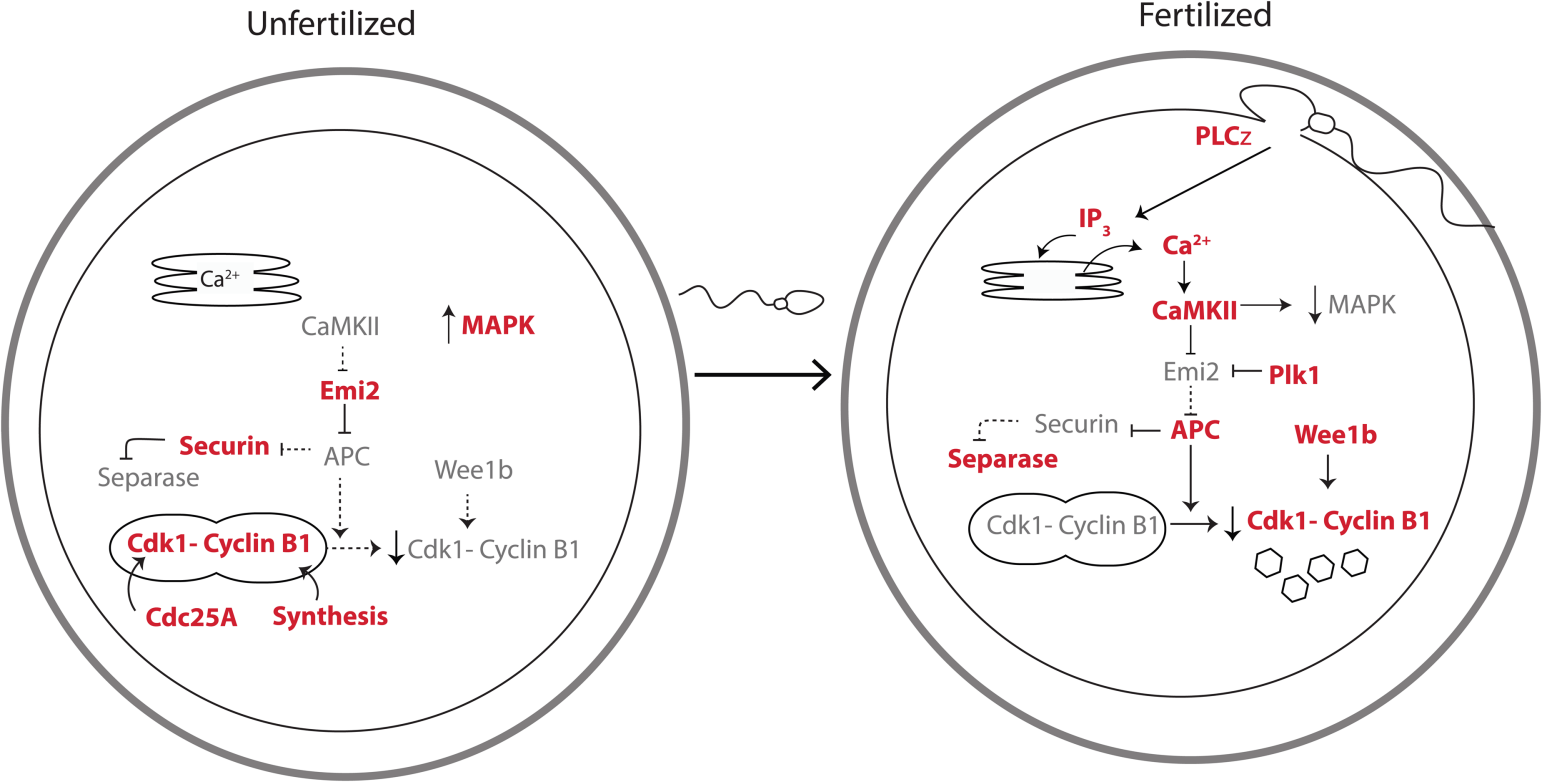
Cell cycle proteins involved in arrest and release from metII during egg activation. During metII arrest, Emi2 is kept high. It inhibits the APC, thus preventing loss Cdk1 activity through cyclin B1 proteolysis. Cdk1 activity is further maintained by continual cyclin B1 synthesis and stimulation of Cdc25. MAPK levels are also high in unfertilized eggs. At fertilization, following sperm-egg fusion, PLCζ diffuses into the egg and hydrolyses PIP_2_ to produce IP_3_ which releases Ca^2+^ from the endoplasmic reticulum and so activates CamKII. Emi2 is a CamKII and Plk1 substrate, with its phosphorylation leading to degradation. Loss of Emi2 frees the APC to be active, causing cyclin B1 degradation and consequently lowering Cdk1 activity, a process enhanced by Cdk1 phosphorylation by Wee1b. APC activation also degrades securin leading to the activation of separase, promoting chromosome segregation by cohesin cleavage. The increase in cystosolic Ca^2+^ also decreases MAPK activity, which promotes the formation of the nuclear envelope.

MI is completed with the extrusion of the first PB and MII entry immediately follows. As the chromosomes remaining in the mature oocyte (hereafter referred to as an egg) are already co-located, it is probably unsurprising that metII is achieved within an hour. The egg becomes arrested at this point until fertilization, and its cell cycle release is a crucial component of fertilization. It is therefore vital that metII arrest and release are tightly controlled (see Figure 2).

During metII arrest, Cdk1 activity is kept high [78]. This is a combination of high levels of cyclin B1 synthesis and an increase in Cdk1 activation through dephosphorylation by the phosphatase Cdc25A [79]. Furthermore, the degradation of cyclin B1 and securin is prevented through the inhibition of APC by Emi2, which appears to be made only at the completion of MI [80]. MetII arrest can last tens of hours, during which time the spindle remains intact, although postovulatory ageing of the egg is observed over extended periods with an associated loss in some spindle integrity [81]. It is not until fertilization that MII is completed and the second polar body is extruded.

At fertilization, the metII egg undergoes a series of physiological and biochemical changes known as activation. When the sperm and egg fuse, phospholipase C zeta (PLCζ) is released from the sperm into the egg cytoplasm [82,83]. This triggers Ca^2+^ release from the endoplasmic reticulum through the hydrolysis of phosphatidylinositol 4,5-bisphosphate (PI(4,5)P_2_) and the production of inositol trisphosphate (IP_3_) [82,83]. The Ca^2+^ release that occurs follows a pattern of oscillations, a series of repetitive, transient increases in cytosolic Ca^2+^ concentrations that last for many hours [82,84,85]. PLCζ activity appears to be sufficient to induce these Ca^2+^ oscillations, which go on to trigger many of the subsequent events of egg activation including resumption of MII. However, there also exists a cryptic secondary factor in the sperm capable of causing some Ca^2+^ release, but is ‘cryptic’ because it is only observable when PLCζ is absent [86,87]. Its presence may be indicative of a backup mechanism, ensuring that fertilization will take place, but its exact identity remains currently unknown.

There is a clear link between the Ca^2+^ oscillations and cell cycle resumption through the Ca^2+^ sensing enzyme calmodulin-dependent protein kinase II (CamKII) [88,89]. CamKII activates protein tyrosine kinase Wee1b, which inhibits Cdk1 through phosphorylation [90]. The APC inhibitor Emi2 is also a CamKII substrate, which leads to further phosphorylation of Emi2 by Polo-like kinase 1 (Plk1) [91]. As Emi2 is degraded, the APC is activated [80], enabling the targeted destruction of cyclin B1 and securin thus promoting cell cycle progression [92]. The centromeric cohesin is no longer protected as Sgo2 is removed and the PP2A phosphatase inhibitor 12PP2A is activated [60,93]. The decrease in securin directly activates separase, which allows the sister chromatids to be separated at anaphase [94]. For the full completion of MII however, the nuclear envelope must reform and chromosomes decondense. This process requires the destruction of another signaling component, MAPK [95]. Like Cdk1, MAPK levels are kept high during metII arrest [96]. However, MAPK requires many Ca^2+^ oscillations before it is destroyed effectively at egg activation [97]. This means that though the destruction of cyclin B1 is sufficient to resume MII, in order to complete MII it is essential that MAPK is destroyed.

Like MI oocytes, metII-arrested eggs are able to respond to DNA damage. However, these eggs respond differently to MI oocytes and the SAC is not activated in response to DNA damage. If metII eggs are exposed to etoposide and nocodazole treatment, only a small amount of Mad1 becomes localized to the kinetochores, far less than that seen in MI oocytes [77]. Furthermore, these damaged eggs are still able to activate at high rates [77]. This shows that similar to somatic cells during mitosis, metII eggs do not display arrest in response to DNA damage. However, very high levels of damage do have a negative effect on metII eggs. Exposing unfertilized metII eggs to the anti-tumor drug doxorubicin (DXRS) triggers apoptosis by the canonical caspase cascade [98]. Crucially, DNA damage can be detected very early in response to DXRS [99]. It is now believed that apoptosis in metII eggs is triggered as a result of two different checkpoints: one is DNA integrity and the other is mitochondrial integrity [100]. Cytochrome *c* leakage from the mitochondria can facilitate the formation of a protein complex known as the apoptosome which then activates caspases [101]. It is important to note that this response to DNA damage only occurs in unfertilized egg. When fertilized, one-cell embryo do not undergo apoptosis in response to drugs such as DXR, instead these cells arrest in their cell cycle much like MI oocytes [98]. The reason for these cell cycle-dependent difference in DNA damage responses remains unknown.

## Conclusions

Despite much of the cell cycle machinery being conserved between mitosis and meiosis, there still appears to be some unique features of control in maturing oocytes and mature eggs. This is likely due to the unique nature of the two meiotic divisions, relating to their prolonged duration punctuated by stops and starts, which likely necessitates for a nunaced level of control not observed in somatic cells. The SAC is active in oocytes yet displays different levels of effectiveness against microtubules attachment errors (weak) and DNA damage (strong arrest). Why this apparent difference in the strength of the SAC exists is unclear, but if resolved may shed some insight into a fundamental aspect of meiotic cell cycle regulation. Similarly, in eggs, the identity of the second sperm-activating factor that is not PLCζ may shed light on a novel signaling pathway employed in eggs to activate the cell cycle machinery required for completion of MII.

## Abbreviations

APC: anaphase-promoting complex
ATM: Ataxia telangiectasia-mutated
CamKII: calmodulin-dependent protein kinase II
DSBs: double-strand breaks
DXRS: doxorubicin
GV: germinal vesicle
I-Mad2: intermediate form of Mad2
IP_3_: inositol trisphosphate
KMN: KNL-1/Mis12 complex Ndc80 complex
LH: luteinizing hormone
MCC: mitotic checkpoint complex
metI: metaphase I
metII: metaphase II
MI: meiosis I
MII: meiosis II
NEB: nuclear envelope breakdown
O-Mad2: open Mad2
PAR: pseudoautosomal region
PB1: first polar body
PI(4,5)P_2_: phosphatidylinositol 4,5-bisphosphate
PLCζ: phospholipase C zeta
Plk1: Polo-like kinase 1
SAC: spindle assembly checkpoint
Sgo2: Shugoshin 2
